# Editorial: Advancements in personalized medicine for head and neck cancer: molecular-based approaches to treatment and care

**DOI:** 10.3389/fonc.2026.1859504

**Published:** 2026-05-15

**Authors:** Mihai Dumitru, Angeles Mercedes Oviedo Santos, Daniela Vrinceanu

**Affiliations:** 1 ENT Department, Carol Davila University of Medicine and Pharmacy, Bucharest, Romania; 2 ENT Department, University Hospital of Gran Canaria Dr. Negrin, Las Palmas de Gran Canaria, Spain

**Keywords:** Advancements, diagnostics, head and neck cancer, healthcare, molecular, personalized

## 1 Introduction

Head and neck cancers encompass a biologically diverse group of malignancies that arise across distinct anatomic subsites yet share a disproportionate impact on speech, swallowing, appearance, and quality of life. Despite major advances in surgery, radiotherapy, and systemic therapy, outcomes remain heterogeneous, reflecting differences in viral oncogenesis, mutational landscapes, tumor microenvironment, and patterns of treatment resistance. Personalized medicine is reshaping this field by moving beyond one-size-fits-all protocols toward molecularly informed prevention, diagnosis, prognostication, and therapeutic selection. Comprehensive genomic profiling, immunophenotyping, and functional biomarkers increasingly enable clinicians to identify actionable alterations (such as receptor amplifications, kinase pathway activation, and fusion events), refine risk stratification, and tailor multimodality care to the individual patient. In parallel, innovative approaches, including immune checkpoint blockade, targeted agents, adaptive de-escalation strategies, and emerging technologies such as radiomics and nanotechnology-enabled drug delivery are expanding the therapeutic window while aiming to limit toxicity. This Research Topic highlights recent evidence across major head and neck subsites, emphasizing how molecular-based insights are translating into practical decision-making, the current limitations of available biomarkers, and the multidisciplinary frameworks required to implement precision oncology responsibly and equitably in routine clinical practice.

## 2 Ear

Primary mucoepidermoid carcinoma of the middle ear is a rare malignancy. Hu et al. describe the case of a 58-year-old woman presented with chronic purulent then bloody otorrhea; mastoidectomy revealed low-grade mucoepidermoid carcinoma confirmed by histology and immunohistochemistry. Initial radiotherapy (3,000 cGy) followed but local recurrence occurred at nine months, prompting subtotal temporal bone resection, partial parotidectomy, and neck dissection with clear margins. Despite salvage surgery, the patient declined further adjuvant therapy, and the patient succumbed to tumor recurrence two years after the second surgery. The report emphasizes diagnostic challenges, the value of early aggressive resection and radiotherapy, molecular testing (MAML2), and lifelong surveillance and multidisciplinary management.

## 3 Nose and nasopharynx

Lu et al. present a 50-year-old man with Epstein–Barr virus–positive nasopharyngeal carcinoma who developed a rare breast metastasis amid disseminated disease. Pathology confirmed metastatic non-keratinizing squamous carcinoma in the breast. After sequential platinum-based chemotherapy and later gemcitabine plus cisplatin combined with the PD-1 inhibitor tislelizumab, the patient achieved a durable partial response and long-term disease control. This case also serves as a reminder of the importance of re-biopsy for recurrent or metastatic lesions. The patient has achieved a survival duration of up to 7 years post-treatment, illustrating treatment responsiveness and the need for individualized, multidisciplinary care.

The study by Li et al. investigates how the traditional Chinese medicine herb pair Hedyotis Diffusae Herba–Scutellariae Barbatae Herba (HDH–SBH) exerts therapeutic effects against nasopharyngeal carcinoma (NPC) by integrating network pharmacology, molecular docking, and in-vitro validation. Researchers identified 36 active compounds and 115 shared targets, with quercetin, luteolin, wogonin, β-sitosterol emerging as key components. Bioinformatics analyses highlighted the PI3K/AKT signaling pathway as the central mechanism, supported by strong predicted binding of core compounds to AKT1, TP53, and BCL2. *In vitro* experiments using 5-8F and CNE2 NPC cell lines showed that HDH–SBH significantly inhibits proliferation and migration, reduces colony formation, and induces apoptosis, accompanied by downregulation of AKT1 and BCL2 and upregulation of TP53. These findings suggest that HDH–SBH suppresses NPC progression primarily by inhibiting PI3K/AKT pathway activation and restoring pro-apoptotic signaling. The authors propose HDH–SBH as a promising multi-target therapeutic candidate, while emphasizing the need for *in vivo* studies, pharmacokinetic profiling, and clinical validation to translate these results into practice.

## 4 Oral cavity and pharynx

A study by Qiu et al. enrolled 385 surgical patients to build and internally validate a nomogram predicting postoperative sleep disturbances. A practical predictive model was developed to identify oral cancer patients at risk of postoperative sleep disturbances. Using PSQI, MDASI-H&N, HADS and SSRS, multivariable analysis identified independent predictors: history of alcohol consumption, longer surgical duration, higher MDASI-H&N and anxiety scores, and lower social support levels. The model showed excellent discrimination (AUCs 0.902–0.967), good calibration, and decision-curve support, offering a clinically useful screening tool for targeted postoperative interventions. Implementation could guide nursing-led screening, personalized interventions, and resource allocation to improve recovery and patient outcomes overall.

Liu et al. describe a 48-year-old man with metastatic gingival squamous cell carcinoma who achieved complete remission after combined immunotherapy, chemotherapy, and radiotherapy, followed by 38 months of pembrolizumab maintenance. Despite sustained disease control, he developed a second primary tumor in the contralateral oral cavity two years after remission, likely influenced by immunoediting, intratumoral heterogeneity, prior radiation exposure, and long-term betel nut and tobacco use. The new tumor was surgically removed, and postoperative S-1 therapy has kept him recurrence-free for nearly two years. The case highlights the need for long-term surveillance even after immunotherapy-induced remission.

Patients undergoing mandibular reconstruction with free fibula flaps for osteoradionecrosis experience significantly worse outcomes than those treated for malignant or benign conditions. They present with greater comorbidity, larger defects, and higher surgical complexity, leading to markedly increased major complications and flap failure. Quality-of-life scores decline sharply postoperatively and show limited recovery, with persistent pain, xerostomia, swallowing difficulty, and impaired social eating. In contrast, benign and malignant cohorts demonstrate clearer functional improvement over 12 months. The findings of Zhang et al. highlight osteoradionecrosis as a distinct, high-risk entity requiring tailored perioperative planning and long-term multidisciplinary support.

The study by Guan et al. evaluates whether adjuvant radiotherapy can be safely omitted in oral squamous cell carcinoma patients who achieve pathologic complete response after neoadjuvant immunochemotherapy. In a matched cohort, survival outcomes were comparable between de-escalation and standard radiotherapy groups, while quality of life was significantly better without radiation. Immune-related toxicities increased due to prolonged PD-1 therapy but remained manageable. Genomic and immune profiling identified TP53 mutations and low B-cell signatures as independent predictors of recurrence, enabling a three-tier risk model. Overall, radiotherapy omission appears feasible for biologically favorable patients, pending prospective validation.

The study by Yang et al. evaluates whether chemoradiotherapy (CRT) offers additional benefit over radiotherapy (RT) alone for oral squamous cell carcinoma patients who achieve a pathologic complete response after neoadjuvant immunochemotherapy. Among 116 patients, including a propensity-matched cohort of 84, CRT did not improve overall survival or locoregional control compared with RT in the general population. However, subgroup analyses revealed that patients with radiologic extranodal extension or poorly differentiated tumors experienced significantly better outcomes with CRT, suggesting persistent high-risk biology despite complete pathological response. In contrast, low-risk patients without these features derived no measurable advantage from CRT and were exposed to substantially higher toxicity. CRT was associated with markedly increased rates of severe mucositis, dermatitis, nausea, hematologic toxicity, xerostomia, fibrosis, and dysphagia. The findings support a risk-adapted adjuvant strategy: reserving CRT for biologically aggressive disease while safely de-escalating to RT alone in lower-risk patients to minimize treatment-related morbidity.

Photodynamic therapy (PDT) for oral squamous cell carcinoma offers a minimally invasive, selective approach that reduces morbidity compared with surgery, radiotherapy, and chemotherapy. Nanotechnology significantly improves PDT by enhancing photosensitizer solubility, tumor targeting, stability, and light absorption through liposomes, polymeric nanoparticles, gold nanorods, quantum dots, and upconversion nanoparticles. These advances according to Esperouz et al. increase ROS generation and treatment precision. However, PDT remains limited by shallow light penetration, tumor hypoxia, photosensitivity, and off-target photosensitizer accumulation. PDT is most effective for superficial or early OSCC and shows promise for oral potentially malignant disorders, with ongoing research aiming to extend its use to deeper or advanced tumors.

Hypopharyngeal squamous cell carcinoma (HSCC) is an aggressive head and neck cancer with poor prognosis, and this study identifies hepatoma-derived growth factor (HDGF) as a key promoter of its malignant behavior. Analysis of TCGA data by Yang et al. shows HDGF is significantly overexpressed in HSCC and correlates with higher grade, advanced stage, alcohol history, lymphovascular invasion, and post-radiotherapy status. Using FaDu HSCC cells, the authors demonstrate that HDGF knockdown markedly suppresses proliferation, colony formation, migration, and invasion. Mechanistically, HDGF depletion reverses epithelial–mesenchymal transition by increasing E-cadherin and reducing N-cadherin, Snail, and Slug. It also inhibits the AKT/mTOR/VEGF pathway, lowering p-AKT, p-mTOR, and VEGFA levels. These findings position HDGF as a central regulator of HSCC progression and a promising therapeutic target, supported by evidence from other cancers where anti-HDGF antibodies show strong antitumor activity.

## 5 Larynx

The study by Huo et al. investigates the biological role of NK4, an antagonist of the HGF/c-Met pathway, in laryngeal squamous cell carcinoma (LSCC) using a TU212 cell model engineered to stably overexpress NK4. Functionally, NK4 overexpression significantly inhibited LSCC cell proliferation, reduced migratory capacity in scratch-healing assays, and suppressed epithelial–mesenchymal transition, as evidenced by downregulation of Snail, Slug, and MMP9 alongside preserved E-cadherin expression. Transcriptomic profiling revealed 320 differentially expressed genes (189 upregulated, 131 downregulated), with enrichment analyses highlighting alterations in biological processes related to cellular regulation, metabolism, and stress responses. Key signaling pathways affected included HIF-1, MAPK, and PI3K–Akt, all central to tumor growth and survival. Overall, the findings demonstrate that NK4 exerts potent tumor-suppressive effects in LSCC by inhibiting proliferation, migration, and EMT while promoting apoptosis, supporting its potential as a therapeutic target.

Patients with recurrent nonmetastatic laryngeal cancer who refused salvage total laryngectomy showed limited benefit from pembrolizumab monotherapy despite uniformly high PD-L1 CPS. In this eight-patient cohort analyzed by Shires et al., over one-third progressed early and died, and most required tracheostomy during treatment. Adding carboplatin and 5-fluorouracil after immunotherapy failure improved outcomes, with 75% of combination-treated patients alive at one year and a median progression-free survival of about ten months. Only one patient achieved a durable response to immunotherapy alone. While total laryngectomy remains the only curative option, chemo-immunotherapy offers better palliative disease control than immunotherapy alone.

The study by Zhao et al. develops and validates a multimodal, decision-level fusion model (DF-Model) to predict postoperative recurrence-free survival in patients with locally advanced laryngeal squamous cell carcinoma. Using data from 278 patients, the authors integrate clinicopathological variables with CT-based radiomics to overcome the limitations of AJCC staging, which lacks biological detail. After feature selection, a Clinic-score and Rad-score are generated and combined through late fusion, outperforming feature-level fusion and single-modality models. In the validation cohort, the DF-Model achieves a C-index of 0.826, showing strong discrimination, calibration, and clinical utility. It enables effective risk stratification across AJCC stages and tumor subsites, identifying high-risk patients who may benefit from intensified postoperative management. Radiomics contributes most of the predictive power, highlighting tumor heterogeneity as a key driver of recurrence. The authors note limitations such as single-center design and call for multicenter prospective validation.

## 6 Salivary glands

The study of Huang et al. profiles 13 Chinese patients with salivary duct carcinoma, revealing a high prevalence of actionable genomic alterations, especially HER2 amplification or activating mutations, which appeared in nearly 70% of cases. TP53 mutations were most common, while MAPK and PI3K pathway alterations were also frequent. Two detailed cases demonstrated meaningful and sometimes durable responses to HER2-targeted therapies, including pyrotinib, trastuzumab, and pertuzumab. Novel HER2 mutations, including exon 16 skipping, were identified and may influence treatment sensitivity. Overall, the work highlights HER2 as a dominant therapeutic target and underscores the value of comprehensive genomic profiling in SDC.

The study by Huang et al. developed and validated a multiphasic CT radiomics nomogram to distinguish atypical parotid carcinomas from pleomorphic adenomas across three medical centers. Using plain, arterial, and venous phase CT scans, the authors extracted radiomics features, selected them through statistical filtering and LASSO, and trained LightGBM classifiers. Arterial-phase features were most informative, and combining radiomics with clinical variables significantly improved diagnostic accuracy. The final nomogram achieved strong performance in internal and external validation, offering better discrimination than clinical assessment alone. This tool provides a non-invasive method to support preoperative decision-making and may reduce unnecessary biopsies while guiding tailored surgical planning.

Radiotherapy-induced xerostomia remains a major burden for patients with head and neck cancer, and this systematic review with Bayesian network meta-analysis evaluated nine non-pharmacological interventions across 30 randomized trials involving 1,595 participants. The analysis by Tai et al. showed that several approaches outperform standard care, but their strengths differ by outcome. Mouthwash provided the most consistent improvement in subjective xerostomia scores, while oral moisturizing gel produced the largest gains in unstimulated salivary flow. Chewing gum ranked highest for stimulated salivary flow, reflecting its dual gustatory and masticatory stimulation. Photobiomodulation demonstrated broad benefits across multiple endpoints, including salivary function. TENS did not significantly change symptoms or flow but ranked best for xerostomia-related quality of life. Safety profiles were comparable across interventions. Overall, the evidence supports a tailored, outcome-specific strategy rather than a single universal therapy, emphasizing symptom-focused, flow-focused, and quality-of-life-focused modalities depending on patient needs.

## 7 Thyroid

Li et al. published a phase II single-arm study which evaluated anlotinib combined with ¹³¹I in iodine-avid distant metastatic differentiated thyroid cancer. Twenty patients received standard-dose RAI plus 12 mg anlotinib in 2-weeks-on/1-week-off cycles. The combination produced a 55% partial response rate and 94.7% disease control, with universal thyroglobulin reduction and no treatment-related deaths. Toxicities were frequent but manageable, mainly hypertension and hand–foot syndrome, with half requiring dose reductions. Patients with cumulative RAI <600 mCi responded better than heavily pretreated patients. Median progression-free survival was not reached at 13.7 months. Overall, the regimen showed promising efficacy, biochemical improvement, and acceptable safety, supporting further randomized trials to validate its role in metastatic DTC.

The report by Guangxu et al. describes an exceptionally rare inflammatory myofibroblastic tumor (IMT) of the thyroid in a 43-year-old woman, notable for a novel ALK point mutation (R395H) identified by next-generation sequencing. Histology showed spindle cells with inflammatory infiltrate, while immunohistochemistry revealed strong ALK-1, TTF-1, PAX-8, and Galectin-3 expression. The ALK-R395H mutation, located in a hotspot region of exon 5, has not been previously reported and may indicate malignant potential. The patient underwent left lobectomy with no recurrence after 37 months. This case expands the molecular spectrum of thyroid IMT and highlights the diagnostic value of combined histologic and genetic analysis.

The study by Zhang et al. investigates how exposure to the plasticizer DEHP may contribute to thyroid cancer (TC) by integrating network toxicology, transcriptomics, survival modeling, and molecular docking. By intersecting DEHP-related targets with differentially expressed genes from TCGA and GEO datasets, the authors identify six shared genes, of which CYP1B1, GABRB2, and TNFSF15 emerge as independent prognostic markers. These genes participate in pathways involving xenobiotic metabolism, synaptic signaling, hormone regulation, and inflammatory responses. A three-gene risk model effectively stratifies TC patients and shows strong predictive performance across datasets. Molecular docking suggests DEHP can directly bind these proteins, supporting mechanistic plausibility. Overall, the study proposes that DEHP disrupts thyroid hormone homeostasis and cellular signaling, promoting TC development and progression.

Dong et al review whether pregnancy is generally safe for women with differentiated thyroid cancer, but optimal outcomes depend on timing, disease status, and careful monitoring. Conception is recommended at least 6–12 months after radioactive iodine therapy, as early pregnancy increases risks such as preterm birth. Thyroid cancer and its treatments may slightly delay fertility, though overall pregnancy rates remain comparable to the general population. During pregnancy, thyroid function and thyroglobulin levels require close surveillance, especially in women with persistent disease. Hypoparathyroidism must be managed proactively to avoid maternal and neonatal complications. Postpartum, recurrence risk modestly increases, and breastfeeding requires planning if further radioactive iodine is needed. Across all stages, multidisciplinary care, psychological support, and individualized reproductive counseling are essential for safe maternal and fetal outcomes.

## 8 Other head and neck tumors

Head and neck squamous cell carcinoma patients receiving neoadjuvant immunochemotherapy show markedly different outcomes depending on the interval to surgery. In a retrospective cohort of 356 patients Seng et al. showed that delaying surgery beyond three weeks significantly reduced both pathologic complete response and major pathologic response rates, while also increasing surgical complications and shortening disease-free survival. Shorter intervals (<3 weeks) preserved higher tumor regression rates without increasing operative risk. Neither p16 status nor the number of neoadjuvant cycles influenced outcomes. Overall, the findings suggest that surgery performed within three weeks after completing neoadjuvant immunochemotherapy may optimize oncologic and perioperative results.

LAT1 is a key amino acid transporter implicated in tumor metabolism and boronophenylalanine uptake for BNCT. In a cohort of 100 HNSCC patients, Cavalieri et al. underlined that high LAT1 expression occurred in 13% of tumors and strongly correlated with hypoxic and mesenchymal transcriptomic subtypes, both associated with aggressive biology and radioresistance. LAT1-high patients had markedly poorer overall survival, with a median of 23 months versus 94 months in LAT1-low cases. Gene-set analyses showed enrichment of hypoxia and glycolysis pathways. These findings position LAT1 as a negative prognostic biomarker and a promising candidate for BNCT-oriented patient selection.

Ga-68 DOTATATE PET/CT demonstrates superior sensitivity for detecting head and neck paragangliomas compared with MRI, MIBG, and FDG PET/CT. It reliably identifies primary, recurrent, and metastatic lesions, including small or multifocal tumors and SDH-mutated disease, where other modalities often miss findings. Across all presented cases, Ga-68 DOTATATE altered management by clarifying disease extent, guiding radiotherapy, avoiding unnecessary surgery, or excluding tumor when other imaging was ambiguous. Despite the small cohort, the study by Alduraibi et al. highlights Ga-68 DOTATATE PET/CT as a highly effective whole-body tool that significantly improves diagnostic confidence and clinical decision-making.

Li et al. retrospectively analyzed 318 patients with glomus jugulare tumors (2003–2022) to identify predictors of postoperative recurrence and to construct a nomogram for individualized risk estimation. Recurrence occurred in 21.4% of patients over a mean 8.7-year follow-up. Multivariate analysis identified younger age, elevated Ki-67 index, negative S-100 expression, and invasion of the facial nerve, internal carotid artery, or intracranial compartment as independent predictors of recurrence. These variables formed the basis of a nomogram demonstrating strong discrimination (AUC: 0.863 training, 0.711 validation, 0.784 test). The model supports stratifying patients into risk categories to guide postoperative management. High-risk individuals may benefit from adjuvant radiotherapy and require long-term surveillance, as recurrence can occur more than a decade after surgery. The authors highlight the need for multicenter validation and incorporation of genetic data to further refine recurrence prediction.

The study by Sun et al. evaluates DEB-TACE using 100–300 μm CalliSpheres^®^ beads combined with PD-1 inhibitors in 31 patients with unresectable head and neck squamous cell carcinoma, showing high short-term efficacy, manageable toxicity, and meaningful quality-of-life improvement. At 1, 3, and 6 months, objective response rates were 96.8%, 87.1%, and 74.2%, respectively, with disease control exceeding 80% throughout follow-up. Median progression-free survival reached 9 months and overall survival 19 months, with a 1-year survival rate of 87.1%. Treatment was generally well tolerated, with most adverse events being grade 1–2, including pain, fever, nausea, fatigue, and hypothyroidism; only three grade 3 events occurred. Patients reported significant improvements in physical and emotional functioning, pain, insomnia, and appetite. Overall, the combined approach appears safe, synergistic, and clinically promising for patients lacking curative options.

## 9 Conclusion

Personalized medicine is increasingly redefining head and neck cancer care by linking tumor biology to prevention, risk stratification, and treatment selection. The studies from this Research Topic highlight how genomics, immune profiling, and radiomics can identify actionable targets, support therapy de-escalation in favorable disease, and guide intensification for high-risk patients. However, biomarker standardization, prospective validation, and real-world implementation remain key hurdles. Future progress will depend on multidisciplinary pathways, access to high-quality molecular testing, and integrated data platforms that translate complex signatures into practical decisions. Ultimately, precision oncology should aim to improve survival while reducing toxicity and preserving function.

